# Supplementary material for: Mechanisms Contributing to the Dysregulation of miRNA-124 in Pulmonary Hypertension
Source: Int J Mol Sci. 2021 Apr 8;22(8):3852. doi: 10.3390/ijms22083852 (PMC8068139; doi:10.3390/ijms22083852)
Supplement: Supplementary file 1 [file ijms-22-03852-s001.pdf]

Table S1: Real-time RT-PCR Primer Sequences

mRNA or pre-miRNA	Forward Primer 5' to 3'	Reverse Primer 5' to 3'
Dorsha	GAGAAGTAACGGTGGAGCTAAG	GGATATGATGGGTCAGAACAGG
DGCR8	GAGAACCCAAGTGAGCCTTT	TAGCTCGGGCAGCTTTATTC
XPO5	GCGTTTCTTCTCTACCCTCTATG	TAGCAAGGTCCTCCACAGTA
Dicer	GTCGTGCCGTATTGGTAGTT	CAGCTCCTCTTGCTCATGTT
TARBP2	AGGAGTATGGGACCAGAATAGG	GTGACCCGGAAGGTGAAATTAG
HPRT	ACGTCTTGCTCGAGATGTGA	AATCCAGCAGGTCAGCAAAG
18S	TGACGGAAGGGCACCACCAG	GCACCACCACCCACGGAATC
Pre-miR-124-1	CAGCGGACCTTGATTTAAATGTCC	CAGCCCCATTCTTGGCATTC
Pre-miR-124-2	ATCAAGATTAGAGGCTCTGCTC	AGTGCAGCCGTAGGCTCC
Pre-miR-124-3	GCGGACCTTGATTTAATGTCT	TTGGCATTCACCGCGTGCCT
Pre-miR-124-total	CTCCGTGTTCACAGCGGACC	CTTGGCATTCACCGCGTG
**MiRNA**	**Forward Primer 5' to 3'**	
miR-124	TAAGGCACGCGGTGAATGC	
Let 7i	CCCCTGAGGTAGTAGTTTGTGCTGTT	
miR-224	CCCTCAAGTCACTAGTGGTTCCGTTTAG	
miR-210-3p	CCCCTGTGCGTGTGACAGCGGCTGA	
miR-210-5p	CCCAGCCCCTGCCCACCGCACACTG	
miR-155	CCCTTAATGCTAATCGTGATAGGGGTT	
U6	CGATACAGAGAAGATTAGCATGGCCC	

Table S2: gRNA sequence used in Figure 6

	target seq	PAM	Location from TSS	strand
sgRNA 1	AGCCGGGGTAATTAACACGG	GGG	-366	+
sgRNA 2	GTGCGTGCGCACTGACAGCG	GGG	-135	-
sgRNA 3	TGAGCACCGTGGGTCGGCGA	GGG	106	+
sgRNA 4	GCACTCCGCAATGCGCTCCC	AGG	391	-
